# Understanding the temporal dimension of the red-edge spectral region for forest decline detection using high-resolution hyperspectral and Sentinel-2a imagery

**DOI:** 10.1016/j.isprsjprs.2018.01.017

**Published:** 2018-03

**Authors:** P.J. Zarco-Tejada, A. Hornero, R. Hernández-Clemente, P.S.A. Beck

**Affiliations:** a European Commission (EC), Joint Research Centre (JRC), Directorate D – Sustainable Resources, Via E. Fermi 2749 – TP 261, 26a/043, I-21027 Ispra (VA), Italy; b Instituto de Agricultura Sostenible (IAS), Consejo Superior de Investigaciones Científicas (CSIC), Alameda del Obispo s/n, 14004 Cordoba, Spain; c Department of Geography, Swansea University, SA2 8PP Swansea, United Kingdom

**Keywords:** Hyperspectral, Red edge, Forest decline, Chlorophyll, Sentinel-2a, Radiative transfer

## Abstract

The operational monitoring of forest decline requires the development of remote sensing methods that are sensitive to the spatiotemporal variations of pigment degradation and canopy defoliation. In this context, the red-edge spectral region (RESR) was proposed in the past due to its combined sensitivity to chlorophyll content and leaf area variation. In this study, the temporal dimension of the RESR was evaluated as a function of forest decline using a radiative transfer method with the PROSPECT and 3D FLIGHT models. These models were used to generate synthetic pine stands simulating decline and recovery processes over time and explore the temporal rate of change of the red-edge chlorophyll index (CI) as compared to the trajectories obtained for the structure-related Normalized Difference Vegetation Index (NDVI). The *temporal trend method* proposed here consisted of using synthetic spectra to calculate the theoretical boundaries of the subspace for healthy and declining pine trees in the temporal domain, defined by CI_time=n_/CI_time=n+1_ vs. NDVI_time=n_/NDVI_time=n+1_. Within these boundaries, trees undergoing decline and recovery processes showed different trajectories through this subspace. The method was then validated using three high-resolution airborne hyperspectral images acquired at 40 cm resolution and 260 spectral bands of 6.5 nm full-width half-maximum (FWHM) over a forest with widespread tree decline, along with field-based monitoring of chlorosis and defoliation (i.e., ‘decline’ status) in 663 trees between the years 2015 and 2016. The temporal rate of change of chlorophyll vs. structural indices, based on reflectance spectra extracted from the hyperspectral images, was different for trees undergoing decline, and aligned towards the *decline baseline* established using the radiative transfer models. By contrast, healthy trees over time aligned towards the theoretically obtained *healthy baseline*. The applicability of this *temporal trend method* to the red-edge bands of the MultiSpectral Imager (MSI) instrument on board Sentinel-2a for operational forest status monitoring was also explored by comparing the temporal rate of change of the Sentinel-2-derived CI over areas with declining and healthy trees. Results demonstrated that the Sentinel-2a red-edge region was sensitive to the temporal dimension of forest condition, as the relationships obtained for pixels in healthy condition deviated from those of pixels undergoing decline.

## 1 Introduction

Forests worldwide are experiencing increasing levels of both abiotic stress, such as drought (Allen et al., 2010), and biotic disturbance (Macpherson et al., 2017, Senf et al., 2017b, van Lierop et al., 2015). Determining the impact of these processes on global vegetation dynamics requires the early detection of changes over time and space. Most of the standard remote sensing methods available are based on the detection of persistent structural changes in canopies due to defoliation processes, which are typical of advanced levels of disturbance in forests. The focus on structure is partly due to the ability to detect seasonal variations in the amount of fractional cover and leaf area index (LAI) over different forest types (Wang et al., 2017) with standard vegetation indices such as the Normalized Difference Vegetation Index (NDVI) (Rouse et al., 1974). This index can be calculated using the spectral bands and bandwidths available in current satellite sensors used for operational monitoring of vegetation (de Moura et al., 2017). While short-term leaf area reductions provide a mechanism for drought tolerance, particularly in isohydric trees (McDowell et al., 2008), sustained defoliation tends to precede die-off (Dobbertin and Brang, 2001), as it might ultimately starve trees of carbon (Galiano et al., 2011). Trees under abiotic stress are also more vulnerable to pathogens, which can cause leaf desiccation and loss of leaf pigments long before they cause defoliation (Wulder et al., 2006). With the magnitude and interaction of abiotic and biotic forest disturbances expected to increase (Seidl et al., 2017), it is thus critical to further develop remote sensing indicators sensitive to progressive manifestations of tree decline to monitor the temporal dynamics of disturbances (Senf et al., 2017a). Apart from revealing structural canopy changes, such indicators should exploit spectral regions linked to specific photosynthetic pigments and thus be sensitive to physiological changes such as chlorosis that might precede leaf area reductions and mortality.

Unfortunately, the heterogeneity of forest canopies complicates the application of the pigment–sensitive indices that have demonstrated utility in uniform crops. Recent research has made significant progress in developing methods that retrieve leaf biochemical constituents and biophysical parameters in the spatial domain (Hernández-Clemente et al., 2014a, Zhang et al., 2008). The retrieval of these photosynthetic traits has been demonstrated, among others, for chlorophyll *a* + *b* (Zarco-Tejada et al., 2001), xanthophylls and carotenes (Hernández-Clemente et al., 2012, Hernández-Clemente et al., 2011), and, more recently, for quantifying chlorophyll fluorescence (Damm et al., 2015), enabling the detection of stressed tree crowns and stands exposed to abiotic stress and diseases (Hernandez-Clemente et al., 2017). Several approaches have been proposed to map variation in plant traits and stress using remote sensing imagery. However, there are few studies in which spatiotemporal data have been validated *in situ* to determine if such indicators are sufficiently robust for the temporal monitoring of early stages of forest decline. To be used in such a way, indicators must, among other things, be resistant to confounding effects generated by background and understory changes (Jonas Lambert, 2013). At the same time, scaling remote sensing-based indicators of canopy state into the temporal domain is critical if such indicators are to be used to identify forest decline processes and distinguish them from natural phenological changes.

The assessment of the temporal dimension of forest decline is potentially feasible through different methods and strategies, namely (i) using time series of spectral vegetation indices related to specific pigments and physiological traits through statistical relationships (Assal et al., 2016, Czerwinski et al., 2014, Vicente-Serrano et al., 2016); (ii) quantifying leaf biochemistry over time using *scaling up* methods (as in Zarco-Tejada et al., 2001 for C_ab_); and (iii) applying radiative transfer model inversions through different retrieval strategies, linking leaf and canopy models (as in Houborg et al., 2007) through wavelet analysis (Ali et al., 2016) and spectral unmixing (Stagakis et al., 2016). In the latter approach, retrievable physiological traits are limited to the input part of both the leaf and canopy models. This can be problematic when specific physiological indicators linked to tree health decline are not included in the radiative transfer models (for example, in the case of xanthophyll pigments currently not modeled in PROSPECT). Each of the approaches listed above has advantages and disadvantages, which depend on the complexity of each method, the heterogeneity of the canopy, the maximum errors allowed in the retrievals, and the spectral and spatial resolution of the data available for the *scaling up* and modeling methods. Although model inversion techniques are considered to be the most advanced, they yield errors of about 10 μg/cm^2^ in the best case scenario for C_ab_ (Hernandez-Clemente et al., 2014b, Yáñez-Rausell et al., 2015), reaching 15–20 μg/cm^2^ and more over heterogeneous canopies. These accuracies are usually acceptable for mapping specific traits related to forest health condition at a given time, but might not be sufficient to detect small changes over time due to the inherent errors involved in the parameter retrievals. From an operational point of view, spectral indices sensitive to specific traits tend to be more robust than parameters retrieved by model inversion, which makes them potentially more suitable for temporal monitoring. This is particularly true when the changes between two consecutive dates are subtle or smaller than the inherent errors of the inversion procedures. However, individual spectral indices tend to be sensitive to multiple leaf and canopy characteristics (e.g., to both biochemical constituents and structural attributes of canopies), making it difficult to disentangle the different expressions of crown stress. Specific strategies are thus needed when using indices in the temporal domain for the monitoring of forest decline.

In this context, narrowband indices have been successfully used over crop canopies to maximize the sensitivity to chlorophyll content while minimizing structural effects. A few examples of such indices are the Photochemical Reflectance Index (PRI) (Gamon et al., 1997) used to track light use efficiency in different forest types (Zheng and Chen, 2017) and other soil-resistant pigment indices such as the Modified Chlorophyll Absorption Ratio Index (MCARI) and the Transformed Chlorophyll Absorption Ratio Index (TCARI) (Haboudane et al., 2002) normalized by the Soil Adjusted Vegetation Index (SAVI, OSAVI) (Rondeaux et al., 1996) to form the TCARI/OSAVI and MCARI/OSAVI indices (Haboudane et al., 2004, Zarco-Tejada et al., 2004). These normalizations are proposed to minimize structural effects on the indices (as in Hernández-Clemente et al., 2011 for xanthophyll pigments). Nevertheless, while these index combinations have proven to work well over uniform and closed canopies, they have failed over heterogeneous forest stands due to the effects of within- and between-crown shadows (Hernandez-Clemente et al., 2014b, Hernández-Clemente et al., 2016). This constraint of index-based pigment estimates is particularly limiting for their use in time series analyses, as diurnal and seasonal changes in within-crown illumination and shadows increase their errors.

In an effort to reduce structure-related artifacts in retrieved plant traits, the red-edge spectral region (RESR) was shown in the 1980s to be sensitive to chlorophyll content while largely unaffected by structural properties (Horler et al., 1983). Since then, the red-edge position has proven to be useful for mapping forest species composition (Zarco-Tejada and Miller, 1999) in closed forest canopies (Hernández-Clemente et al., 2016) and conifer forests (Zarco-Tejada et al., 2004) due to its sensitivity to chlorophyll irrespective of crown shadows. These earlier studies involving the RESR were carried out using hyperspectral data and later evaluated with MERIS on board ENVISAT as the first attempt to use RESR parameters for forest monitoring from a satellite sensor (Hu et al., 2008). However, it is well known that both chlorophyll and structure play a role in the shape and temporal dynamics of the RESR (Curran et al., 1990). Therefore, the red-edge chlorophyll index (CI) (e.g. R_750_/R_710_ as in Zarco-Tejada et al., 2001) will respond to changes in C_ab_ but will be largely affected by LAI.

With the launch of the MultiSpectral Imager (MSI) on board Sentinel-2a in 2015 and Sentinel-2b in 2017, there is a potential opportunity to use the RESR at 20 m spatial resolution and 18–19 nm full-width half-maximum (FWHM) to estimate chlorophyll and nitrogen content in vegetation (Clevers and Gitelson, 2013), among other biophysical parameters (Frampton et al., 2013); (Castillo et al., 2017), and to monitor forest condition based on such parameters. In this study, we explored the temporal dynamics of forest decline using the RESR obtained from both high-resolution hyperspectral imagery and Sentinel-2a satellite data. In particular, we propose a methodology using the RESR to understand the effects of physiological and structural changes over time and reveal how pixels of trees under decline deviate from the trajectories of pixels of trees in healthy condition. The study had a threefold objective. Firstly, we evaluated the RESR for conifer forest crown condition monitoring in the temporal dimension, assessing the temporal trajectories of chlorophyll- and structure-sensitive indices as indicators of the variation over time of both chlorophyll and crown LAI in individual pine trees. Secondly, we assessed the forest decline process in the temporal domain using a 3D radiative transfer model, accounting for changes in both crown C_ab_ and LAI over time and their temporal effects in the red-edge spectral region. Thirdly, we validated the proposed methodology using three high-resolution hyperspectral flights conducted within a two-year period along with field assessments of 400 trees for defoliation and chlorosis. Finally, the methodology proposed here, named the *temporal trend method*, was applied to Sentinel-2a images acquired at the same time as the hyperspectral datasets and the field evaluation of forest health status to explore whether the Sentinel-2 red-edge bands and the proposed method were sensitive to the decline processes under study.

## 2 Materials and methods

### 2.1 Study site and field data collection

The study site comprised a 7000 ha pine forest in the region of Extremadura, Spain (40°18′N, 6°6′W) at elevations between 370 and circa 1000 m.a.s.l. with both mixed stands and stands dominated by a single species. The predominant species was *Pinus pinaster*, with *Pinus nigra* mostly growing at higher elevations. Most of the pine stands in the area were planted in the 1960s and thinned in 2009. A total of four field campaigns were carried out to assess the forest condition within the study site, based on the health of a sample of trees that had attained or had the potential to attain maximum height and crown development. *Pinus pinaster* trees were sampled to cover the full range of tree conditions and scored between 0 and 5 on three metrics: (i) percent of defoliation, comprising smaller branches and branchlets with missing needles, needles shed prematurely, and dry needles with brown or reddish condition; (ii) percent of discoloration, referring to color anomalies in live needles, i.e. chlorosis; and (iii) canopy die-off, covering large dead branches that have lost their needles and sprouts. A score of 0 was assigned to trees without any sign of declining health, with other scores reflecting the percentage of the crown displaying poor health; level 1 = [1–25%], level 2 = [26–50%], level 3 = [51–75%], level 4 = [76–99%], level 5 = 100%. In addition, diameter at breast height (DBH), projected crown size and height were measured for each tree. Field campaigns were conducted in 2014/11, 2015/11, 2016/02, and 2016/06 (Table 1). Foresters of the local authorities surveyed 252 pine trees in the first field assessment (2014/11) and 411 trees in the second evaluation (2015/11). Changes in forest condition were assessed for each of these 411 trees recording changes in crown health between November 2015 and June 2016, labeling each tree as either ‘healthy’ or ‘in declining health’ between both dates.

**Table 1:** Flight dates and field evaluations conducted during 2014, 2015, and 2016 airborne and field campaigns.

Year	Field evaluation period	Flight dates hyperspectral	Date Sentinel-2	Trees evaluated	Field assessment^a^
2014	Nov 2014-Jan 2015	28/Jan/2015	–	252	DEFO, DISC, DIE
2015	Nov-Dec. 2015	2/Dec/2015	29/Nov/2015	411	DEFO, DISC, DIE
2016	Feb. 2016			411	DEFO, DISC, DIE
	June-Aug. 2016	20/Jun/2016	19/June/2016		Tree condition/relative change^b^

Level 0 = trees without any sign of declining health; level 1 = [1–25%], level 2 = [26–50%], level 3 = [51–75%], level 4 = [76–99%], level 5 = 100%.

a DEFO = percent of defoliation; DISC = percent of discoloration; DIE = canopy die-off.

b The 411 trees were ranked for their relative change in tree condition between Nov. 2015 and June 2016.

### 2.2 Airborne hyperspectral campaigns & Sentinel-2 datasets

Three airborne campaigns were conducted on 28/01/2015 (Flight 1), 02/12/2015 (Flight 2) and 20/06/2016 (Flight 3) (Table 1). The correspondence between the field surveys and the field evaluations was warranted to make proper comparisons: Flight 1 (January 2015) was related to the conditions of trees assessed in winter 2014 field campaign (2014/11), particularly because the changes during winter are minimal. The Flight 2 (December 2015) was related to the conditions of trees evaluated in winter 2015 field campaign (2015/11). Finally, Flight 3 (June 2016) was related to the conditions of trees evaluated in spring 2016 field campaign (2016/06).

The flights were conducted using a micro-hyperspectral imager (Headwall Photonics, Fitchburg, MA, USA) on board a Cessna aircraft operated by the Laboratory for Research Methods in Quantitative Remote Sensing (QuantaLab), Consejo Superior de Investigaciones Científicas (IAS-CSIC, Spain). The hyperspectral camera was flown with the heading of the aircraft on the solar plane at 400 m above ground level (AGL) at 12:00 GMT providing a swath of 380 m at 40 cm pixel resolution. The acquisition and storage module achieved a 50 fps frame rate with integration time set to 18 ms. The 8 mm focal length lens provided an instantaneous field of view (IFOV) of 0.93 mrad and an angular field of view (FOV) of 50°. The hyperspectral images were collected in the 400–885 nm region with 260 bands acquired at 1.85 nm/pixel and 12-bit radiometric resolution yielding a 6.4 nm FWHM with a 25–µm slit. The imagery acquired by the micro-hyperspectral sensor was orthorectified and radiometrically calibrated as described in (Zarco-Tejada et al., 2016). Atmospheric correction was carried out using the SMARTS model (Gueymard, 1995, Gueymard, 2001) with aerosol optical depth measured at 550 nm with a Micro-Tops II sunphotometer (Solar LIGHT Co., Philadelphia, PA, USA) as was done in previous studies (Berni et al., 2009, Calderón et al., 2013, Calderón et al., 2015, Zarco-Tejada et al., 2012).

**Fig. 1:**
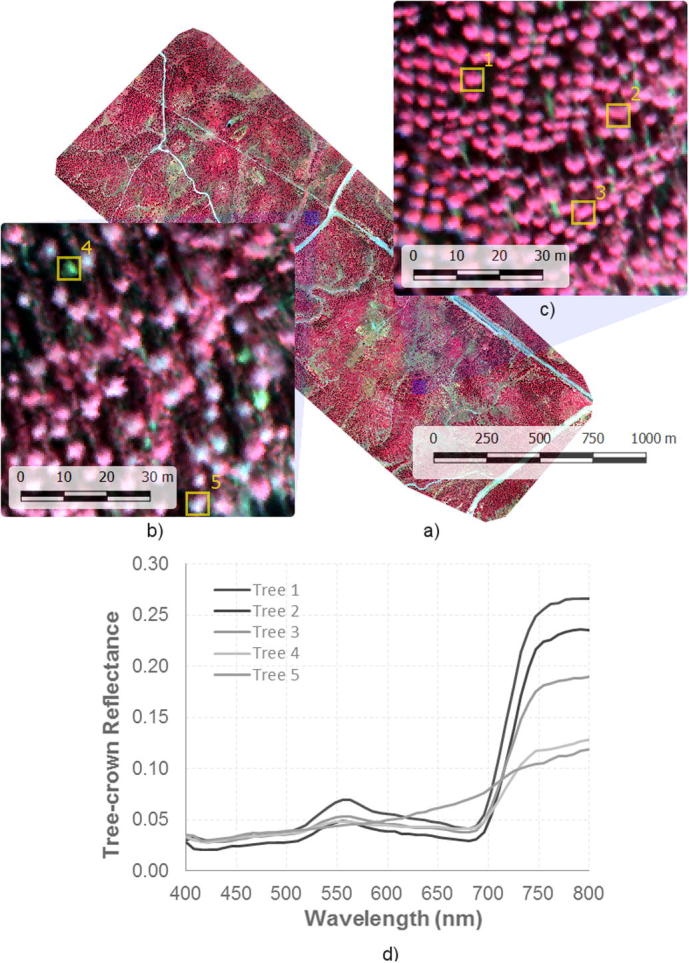
(a) Airborne hyperspectral image acquired with the micro-hyperspectral imager at 40 cm resolution. Zoom-in on (b) an area with widespread tree health decline (decline level = 5), and (c) an area with trees in healthy condition (decline level = 0), and (d) hyperspectral reflectance extracted from trees sampled in each of these areas (trees 1–5).

The high-resolution imagery acquired with the hyperspectral camera (Fig. 1) enabled the identification of pure crowns over the entire scene using automatic object-based crown detection algorithms. Fig. 1 shows a zoom with an example of two areas with low (trees 1–3) and severe level of decline (trees 4, 5). The spatial resolution of the image also enabled the discrimination of shaded and sunlit soil and crown components, minimizing the effects due to the background and within-crown shadow on the border pixels of each tree crown. The object-based image segmentation methods were based on both Niblack’s thresholding method (Niblack, 1986) and Sauvola’s binarization techniques (Sauvola and Pietikäinen, 2000) to separate tree crowns from the soil. Next, we conducted a binary watershed analysis using the Euclidian distance map for each object to automatically separate trees with overlapping crowns. The pure-crown reflectance (Fig. 1) extracted from the hyperspectral imagery was used to calculate the hyperspectral indices described in the following section.

**Fig. 2:**
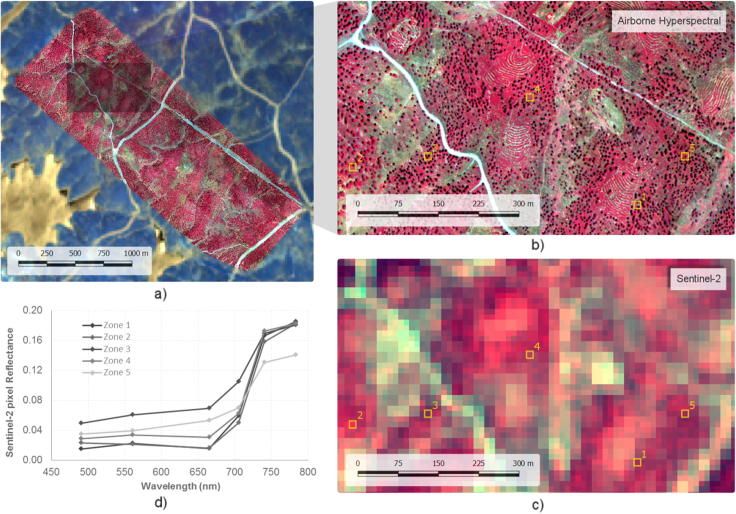
Hyperspectral mosaic overlaying the Sentinel-2a scene (a) including a zoom over a smaller area showing the spatial resolution of both the high-resolution hyperspectral image (b) and the Sentinel-2a scene (c). The Sentinel-2a spectral signature of pixels located over trees with different health decline levels is shown in (d).

The MultiSpectral Imager on board Sentinel-2a acquires imagery at ten-day intervals at the equator under constant viewing conditions. The Sentinel-2a images of the study area used were those obtained between July 2015 and February 2017. The revisit frequency over the area increased due to overlap between swaths from contiguous orbits, providing a larger data set to select scenes free from haze and clouds. The MSI on board Sentinel-2 yields images at 12 bits in 13 spectral bands at different spatial resolutions: four bands at 10 m (central wavelengths at 496.6, 560.0, 664.5 and 835.1 nm with a bandwidth of 98, 45, 38 and 145 nm, respectively), six bands at 20 m (central wavelengths at 703.9, 740.2, 782.5, 864.8, 1613.7 and 2202.4 nm with a bandwidth of 19, 18, 28, 33, 143 and 242 nm, respectively) and three bands at 60 m of spatial resolution (central wavelengths at 443.9, 945.0 and 1373.5 nm with a bandwidth of 27, 26 and 75 nm, respectively). An atmospheric correction was applied to all non-cloudy Top-Of-Atmosphere (TOA) Level-1C pixels, which included a cirrus cloud correction (Richter et al., 2011). Level-2A products were generated within Sen2Cor software, version 2.3.1. The processing chain from Level-0 to Level-1C was performed by the Instrument Data Processing (IDP) functionality of the Payload Data Ground Segment (PDGS) (Fig. 2).

### 2.3 Simulating the temporal effects of forest decline in the red-edge spectral region using the FLIGHT 3D model

A simulation analysis was conducted to assess the confounding temporal effects of defoliation and chlorosis in the context of forest decline at the tree crown level. Radiative transfer modeling was applied using the leaf optical PROperties SPECTra (PROSPECT-5) model (Feret et al., 2008, Jacquemoud and Baret, 1990) coupled with the three-dimensional (3-D) ray-tracing model (FLIGHT) (North, 1996) to simulate tree crown and canopy reflectance in the observation direction as a function of field components.

In particular, the PROSPECT-5 model was used to simulate leaf reflectance and transmittance of the mesophyll layer parameterized by chlorophyll content (C_ab_) in µg/cm^2^, leaf dry mass per unit area (C_m_) in mg/cm^2^, leaf water mass per unit area (C_w_) in mg/cm^2^, and effective number of leaf layers (N) (Jacquemoud and Baret, 1990). The model was originally applied to broadleaf vegetation, but has since been validated to simulate the optical properties of conifer needles (Hernandez-Clemente et al., 2014b, Moorthy et al., 2008, Morsdorf et al., 2008). By contrast, the 3-D FLIGHT model follows Monte Carlo techniques to calculate directional reflectance within crown boundaries and deterministic ray tracing between the crowns and other canopy scene components. In the FLIGHT model, canopy reflectance is simulated by accumulating photon energy in the observation direction as a function of different forest canopy components. It defines the canopy structural heterogeneity from inputs such as crown shape and size, tree height, position, density and distribution, and background reflectance. The nominal values and entire set of input parameters and ranges for the leaf (PROSPECT) and canopy (FLIGHT) modeling strategy used in this study are summarized in Table 2.

**Table 2:** Inputs used in this study for the PROSPECT + FLIGHT simulations: nominal values and range of variation used in the simulation analysis.

Variable	Variable code	Nominal values	Range
*PROSPECT-5*			
Mesophyll structure	N	2.1	–
Chlorophyll content	C_ab_ (μg/cm^2^)	35	10–70
Carotenoid content	C_x+c_ (μg/cm^2^)	12	2–25
Water content	C_w_ (mg/cm^2^)	0.013	–
Dry matter	C_dm_ (mg/cm^2^)	0.024	–
Senescent material	C_s_	0	0

*FLIGHT*			
Solar zenith, view zenith (°)	θ_s_, θ_v_	31.3, 0.0	–
Solar azimuth, view azimuth (°)	Φ_s_, Φ_v_	30.44, 0.0	–
LAI		3.15	0–5
Leaf angle distribution	LAD[1–9]	0.015, 0.045, 0.074, 0.1, 0.123, 0.143, 0.158, 0.168, 0.174	
Crown shape	C_s_	Cones	
Crown radius	C_r_	4.5	
Minimum and maximum height to first branch (m)	H_min_, H_max_	4.0, 10.0	
Soil reflectance	ρ_λsoil_	Image-based	
Soil roughness	Θ_soil_	0	
Solar irradiance	ρ_λs_	ASD measurements	

**Fig. 3:**
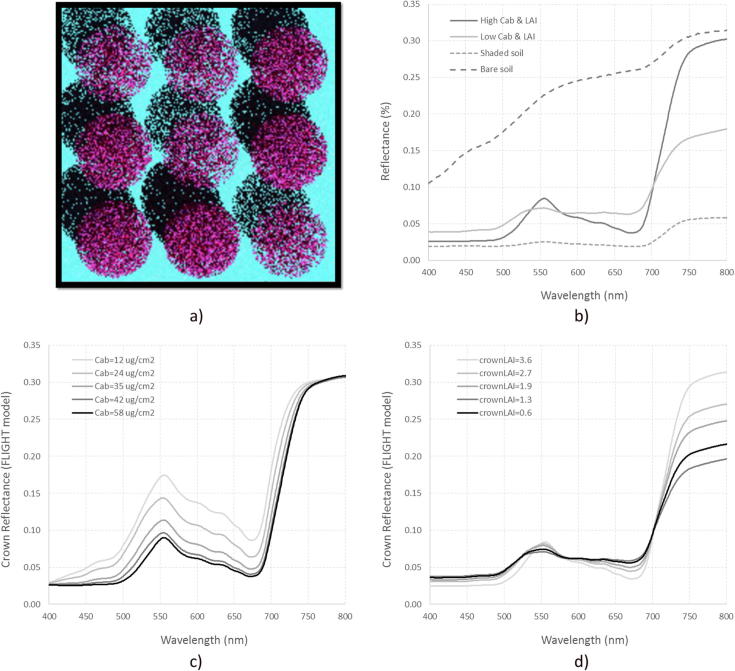
FLIGHT 3D scenes of the study areas (a) and spectra simulated from scene components (b). Simulations conducted for C_ab_ variation between 10 and 60 μg cm^−2^ (c) and for crown LAI values between 0.5 and 4 (d).

The PROSPECT + FLIGHT modeling strategy described above was used to define a complete set of simulation experiments to understand the temporal dynamics of the entire RESR (i.e., spanning the red, red-edge, and NIR regions) as trees gradually decline in health over time. The particular objective of the simulations was to assess the temporal trajectories and rate of change in the time domain occurring in the red, red-edge, and NIR regions due to C_ab_ and crown leaf area index (cLAI) variation. Such traits are normally evaluated by visual field inspections as part of forest health assessment protocols. Here, a total of 5000 3D simulations were conducted with varying inputs of C_ab_ and cLAI, indicators of respectively chlorosis and defoliation (Table 2), mimicking the architecture of the study area described above: a sparse coniferous landscape with large background and understory influence (Fig. 3).

**Fig. 4:**
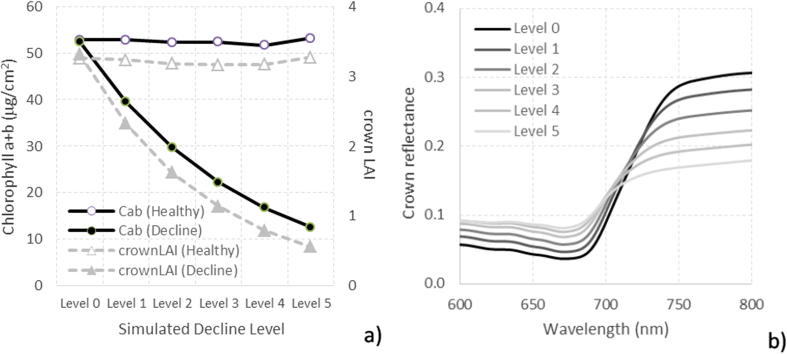
Temporal simulation of crown reflectance for a healthy tree (decline level 0) entering into decline (decline levels 1–5) by gradually decreasing C_ab_ and crown LAI (inputs shown on the left plot) (a) and their effects on crown reflectance (b).

A first set of simulations consisted of C_ab_ and cLAI decreasing simultaneously to model the temporal trend experienced by a healthy coniferous tree that gradually declines. We started from its initial healthy condition with high cLAI and C_ab_ values (labeled here as ‘level of decline = 0’) passing sequentially through increasing levels of damage (decline levels 1–5) by reducing both cLAI and C_ab_ over time (Fig. 4). In this first set of simulations, crown reflectance was also modeled for a tree that kept its biochemical and crown LAI inputs constant over time (labeled as ‘healthy’ in Fig. 4a) and for a gradually declining tree in which both C_ab_ and crown LAI decreased from 60 to 10 µg/cm^2^ and from 4 to 0.5, respectively (decline level = 5, labeled as ‘advanced decline’) (Fig. 4a). The temporal effects observed in the RESR due to increasing decline levels for a pine tree simulated with the structural characteristics of the study area are shown in Fig. 4b.

**Fig. 5:**
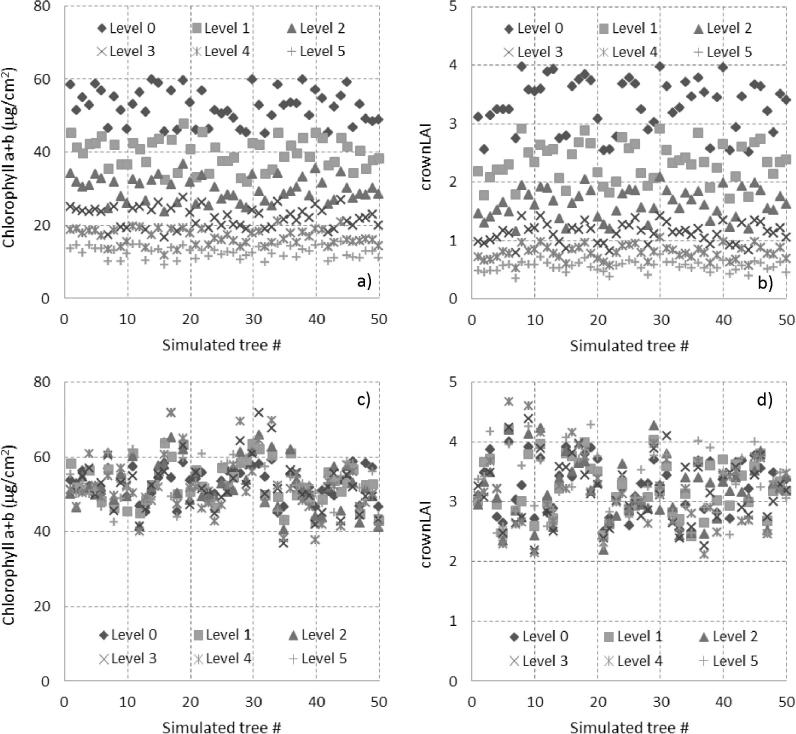
Inputs used for the simulation of decline levels over time: 50 trees starting in healthy condition (Healthy, Level 0) in which C_ab_ (a) and crownLAI (b) are reduced randomly by 20–30% over 5 decline levels (decline levels 1–5); as a control, another 50 trees were kept randomly changing ±10% without decline, simulating healthy levels over time for both C_ab_ (c) and crownLAI (d).

A second set of simulations replicated 50 different trees that started in *healthy* condition (decline level 0) and went into decline with time, experiencing a decrease between 20% and 30%, randomly drawn from a uniform distribution, in both C_ab_ (Fig. 5a) and cLAI (Fig. 5b) with each successive level of decline (levels 1–5). As a control, another 50 simulated trees were allowed to change only within ±10% without progressive decline, as a proxy for the natural variability in tree condition over time (Fig. 5c and d). The purpose of this second set of simulations was to mimic the natural, non-directional, temporal variation in tree crown condition, manifested as random temporal changes of both C_ab_ and cLAI, and compare its effects on the temporal changes in the RESR to those of sustained decreases in crown condition.

A third set of simulations was used to evaluate not only the decline processes in both C_ab_ and cLAI and their effects on the RESR in the temporal domain, but also the recovery process over time. The inputs were selected to simulate a healthy tree that goes through 11 stages simulating a gradual temporal decrease of both cLAI and C_ab_, followed by a full recovery after the maximum values for chlorosis and defoliation are reached. In this set, decoupling the structural and pigment degradation changes was accomplished by decreasing C_ab_ over time while cLAI was kept unchanged, and in turn, by decreasing cLAI over time while keeping C_ab_ unchanged.

The simulated temporal effects of decline and recovery processes were evaluated using red-edge and NIR indices generally accepted as suitable for decline detection. They were calculated from the simulated synthetic spectra for all scenarios and decline levels. In particular, four pigment-related indices and a structure-related index widely used for vegetation monitoring were calculated: (i) the red-edge CI sensitive to C_ab_ and robust to crown shadows in forest areas (Zarco-Tejada et al., 2001); (ii) the TCARI chlorophyll index normalized by OSAVI to minimize changes in LAI (Haboudane et al., 2002); and (iii) the Macc index designed as a CI that is robust to directional effects (Maccioni et al., 2001). Although the PRI index is not calculated in the red-edge spectral region, it is sensitive to the de-epoxidation state of the xanthophyll cycle pigments (Gamon et al., 1997) and proposed for stress detection. Considering this, it was also calculated from the synthetic spectra to evaluate the temporal effects of both C_ab_ and cLAI on the index. Finally, the widely used NDVI structural index (Rouse et al., 1974) sensitive to LAI variation was also calculated. Table 3 shows the equations and references for the indices used in this study.

**Table 3:** Structural and chlorophyll indices used in this study.

Index	Equation	Reference
NDVI	NDVI = (R_800_ − R_670_)/(R_800_ + R_670_)	Rouse et al. (1974)
Red Edge CI	CI = R_750_/R_710_	Zarco-Tejada et al. (2001)
TCARI/OSAVI	TCARI/OSAVI = [3 * [(R_700_ − R_670_) − 0.2 * (R_700_ − R_550_) * (R_700_/R_670_)]]/[(1 + 0.16) * (R_800_ − R_670_)/(R_800_ + R_670_ + 0.16)]	Haboudane et al. (2002)
PRI	PRI = (R_570_ − R_531_)/(R_570_ + R_531_)	Gamon et al. (1992)
Macc	Macc = (R_780_ − R_710_)/(R_780_ − R_680_)	Maccioni et al. (2001)

The indices assessed in this study have demonstrated different degrees of sensitivity to chlorophyll content variation under diverse scenarios and canopy types. Nevertheless, they are differently affected by background, shadows and canopy structure. They have therefore shown various levels of success depending on the canopy type, differing between natural vegetation (Demarez and Gastellu-Etchegorry, 2000, Moorthy et al., 2008) and agricultural crops (Haboudane et al., 2001, Rondeaux et al., 1996, Viña et al., 2011) due to the diverse heterogeneity levels. In the context of the present study focused on pine forest areas, the background (i.e., understory) effects on the indices were assessed with FLIGHT simulations of pine stands. In particular, the effects caused by the understory on the relationships between each vegetation index and C_ab_ were evaluated using three different soil types extracted from the hyperspectral image. The different indices were then assessed for their sensitivity to both C_ab_ and cLAI as a function of the temporal change experienced by trees entering into decline or recovering from decline.

The method proposed in this study consists of evaluating the temporal rate of change for the NDVI (representing structure) as compared to the temporal rate of change for each of the pigment indices (PVI) selected (CI, TCARI/OSAVI, Macc and PRI). In particular, the method calculates the temporal rate of change for each PVI vs. NDVI in the form PVI_time=n_/PVI_time=n+1_ vs. NDVI_time=n_/NDVI_time=n+1_ in order to determine the temporal trajectories of a pair of indices of which one is mostly sensitive to structure while the other is mostly sensitive to pigment content. The different temporal rate of change obtained for structural vs. pigment-related indices is used to build the temporal trajectories in order to understand if a tree crown is gradually increasing or decreasing its decline level, or if it is maintaining a healthy condition over time.

The method proposed here was applied first to the simulated spectra to derive conclusions on the temporal trajectories of the synthetic indices. Next, the method was applied to the real data extracted from the very high resolution hyperspectral imagery acquired over the study sites in January 2015, December 2015 and June 2016. Finally, the methodology was assessed with real Sentinel-2a data using the red, red-edge and NIR bands available. The method was validated using the temporal ground truth data collected that was coincident with the remote sensing imagery, by comparing the trajectories obtained from the hyperspectral and Sentinel-2a imagery with the assessment of decline made in the field for 411 individual trees.

## 3 Results and discussion

### 3.1 Field data analysis

**Fig. 6:**
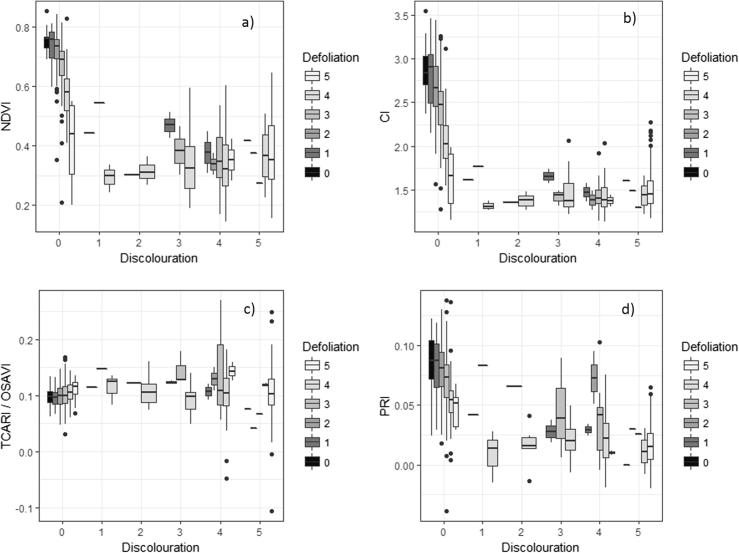
Selected spectral indices derived from hyperspectral images vs. tree crown discolouration and defoliation as assessed during field campaigns. Data from field campaigns in 2014/11 and 2015/12 are compared with images collected on 2015/01/28 and 2015/12/02, respectively, for the NDVI (a), CI (b), TCARI/OSAVI (c) and PRI (d). Sample sizes (n_level_) for discoloration levels are: n_0_ = 410, n_1_ = 5, n_2_ = 8, n_3_ = 16, n_4_ = 62, n_5_ = 162; those for defoliation levels are: n_0_ = 27, n_1_ = 24, n_2_ = 106, n_3_ = 215, n_4_ = 124, n_5_ = 167. Level 0 corresponds to trees without any sign of declining health, while other scores reflect the percentage of the crown displaying poor health: level 1 = [1–25%], level 2 = [26–50%], level 3 = [51–75%], level 4 = [76–99%], level 5 = 100%.

The statistical analysis conducted between the field evaluations of tree health condition and the spectral indices calculated from the pure-crown airborne hyperspectral data showed that unhealthy trees were characterized by a large variability in terms of discoloration in most indices, such as the NDVI, red-edge CI, TCARI/OSAVI and PRI (Fig. 6). The NDVI and CI showed particularly high differences in the absolute values between healthy trees (level 0) and declining trees (levels 1–5).

**Fig. 7:**
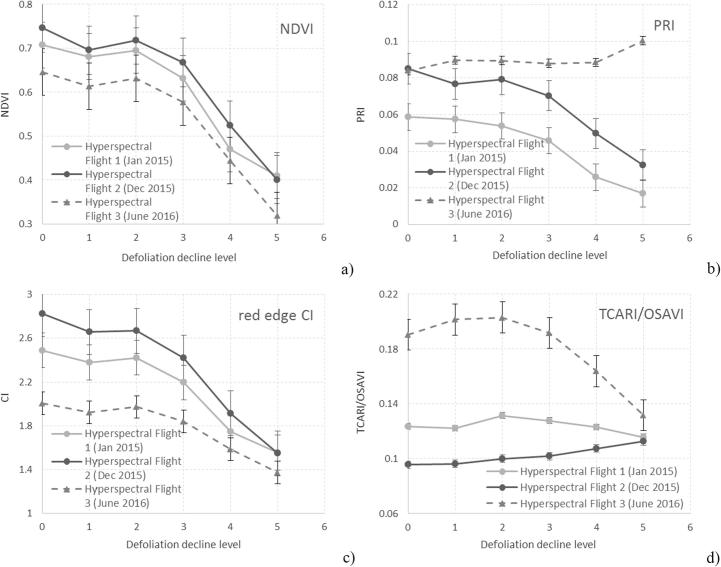
Response of the NDVI (a), PRI (b), red-edge CI (c), and TCARI/OSAVI (d) to crown defoliation decline levels using hyperspectral data from the three airborne campaigns conducted in this study. Patterns are indicative of reflectance effects of structural canopy changes associated with canopy health decline. Symbols indicate mean values across trees and whiskers indicate standard deviations. Level 0 corresponds to trees without any sign of declining health, while other scores reflect the percentage of the crown displaying poor health: level 1 = [1–25%], level 2 = [26–50%], level 3 = [51–75%], level 4 = [76–99%], level 5 = 100%.

As expected, all indices evaluated in this study, including those influenced primarily by chlorophyll content, were sensitive to canopy decline when calculated from the three hyperspectral flights carried out in this study with one exception (Fig. 7): the PRI (Fig. 7b), which showed a divergent response to decline on one date. Interestingly, the different trend of the PRI was recorded by the flight carried out in summer (Flight 3) as opposed to the winter flights (Flight 1 and Flight 2) probably due to the downregulation of photosynthesis activity and the xanthophyll cycle effects caused by drought stress in summer. By contrast, the other indices showed rather consistent trends across the three flight times of January 2015, December 2015, and June 2016. Both the NDVI (Fig. 7a) and CI (Fig. 7c) indices showed consistent trajectories as a function of decline.

### 3.2 Modeling results

**Fig. 8:**
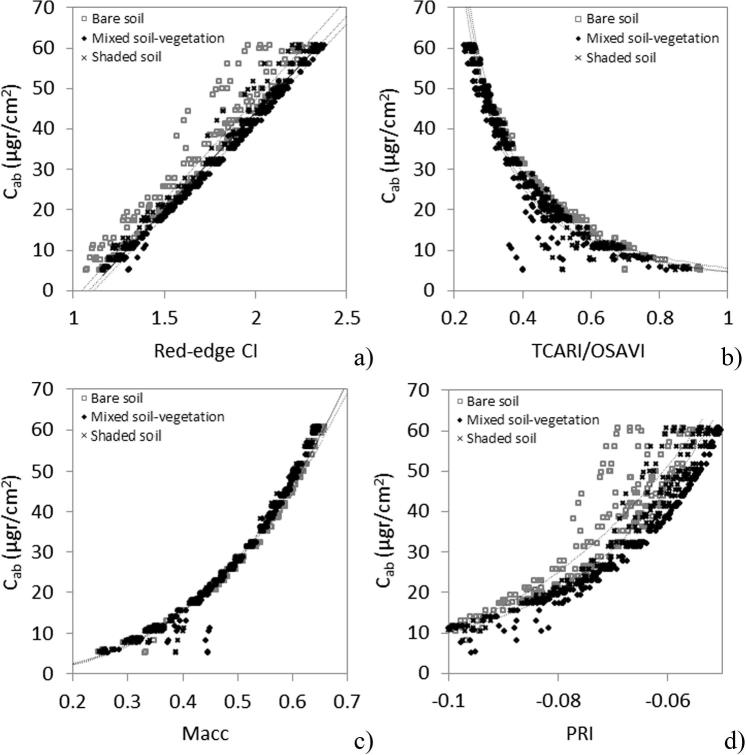
Simulated vegetation indices vs. C_ab_ using the PROSPECT + FLIGHT models with inputs from the study site, evaluating the effects of three soil background spectra from the study area on the red-edge CI (a), TCARI/OSAVI (b), Macc (c) and PRI (d) indices used for C_ab_ quantification. Line fits have the intention to show the displacement between each data series.

Relationships between tree-level reflectance and chlorophyll content for simulated trees using the PROSPECT and FLIGHT models with structural inputs representing the study area are shown in Fig. 8 for the CI (Fig. 8a), TCARI/OSAVI (Fig. 8b), Macc (Fig. 8c) and PRI (Fig. 8d). The three different soil spectra used as input for the simulations were extracted from the hyperspectral image acquired in January 2015 over the site. Of the indices evaluated, the PRI was the most affected by soil background variations while the red-edge CI showed the most linear response to C_ab_.

**Fig. 9:**
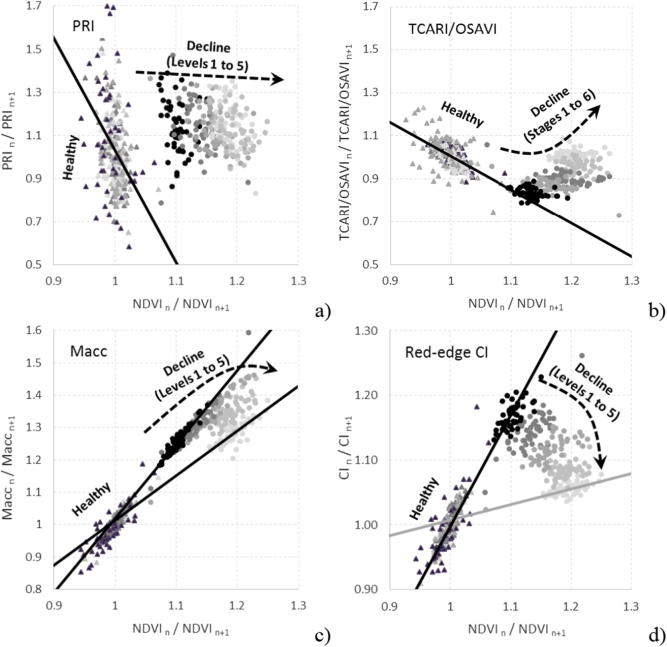
Change rates in the PRI (a), TCARI/OSAVI (b), Macc (c), and red-edge CI (d) indices vs. the NDVI, all derived from synthetic spectra. Rates are calculated for consecutive time steps for healthy trees (triangles) and for trees in declining health progressing through five levels (dots of decreasing darkness). Panel (d) shows that in the CI vs. NDVI temporal change rate, trees in declining health were bound by two baselines and progressed between them, while healthy trees concentrated and remained at the intersection of those baselines over time. Triangles (dots) show the simulated healthy (decline) trees. Decline stages are shown in grey levels from black (low decline) to light grey (high decline). Black and grey lines indicate the boundaries that establish the baselines for healthy trees and trees in severe decline, respectively.

The use of the *temporal trend method* to calculate the change rates in the pigment indices vs. the NDVI structural index from the synthetic spectra can be observed in the PRI (Fig. 9a), TCARI/OSAVI (Fig. 9b), Macc (Fig. 9c) and red-edge CI (Fig. 9d). The modeling results obtained when simulating healthy and increasingly unhealthy trees over time suggest that, in the four cases, healthy trees (decline level 0, labeled as ‘healthy’) were concentrated within the same region, while trees undergoing decline moved away from that region as decline increased over the course of the temporal simulations (decline levels 1–5). Although these four index pairs (i.e., NDVI vs. CI, NDVI vs. Macc, NDVI vs. TCARI/OSAVI and NDVI vs PRI) are of potential interest to detect decline processes, we further explored the case of the CI vs. the NDVI (Fig. 9d) because of (i) its direct applicability to both hyperspectral and Sentinel-2 imagery, therefore of interest for operational purposes; (ii) its greater potential for modeling the effects of the discoloration and defoliation produced by forest decline compared to the other vegetation indices, given that both indices are based on the RESR and have been used successfully to monitor vegetation in other studies; and (iii) although the four index pairs show potential for the applicability of this methodology, the temporal trends as a function of decline levels observed in the CI vs. NDVI pair (Fig. 9d) shows a subspace delimited by two boundary lines (baselines) that can be potentially used to define decline levels.

**Fig. 10:**
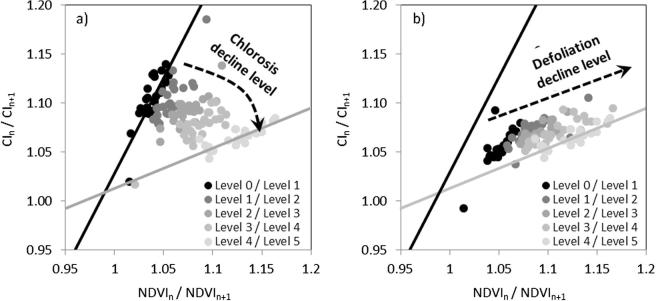
Simulations carried out to assess the changes in only chlorosis and only defoliation over time regarding the rate of change of the CI vs. the NDVI with fixed LAI (a) and fixed C_ab_ (b). Dots represent individual simulated trees. Black and grey lines indicate the boundaries that establish the baselines for healthy trees and trees in severe decline, respectively.

**Fig. 11:**
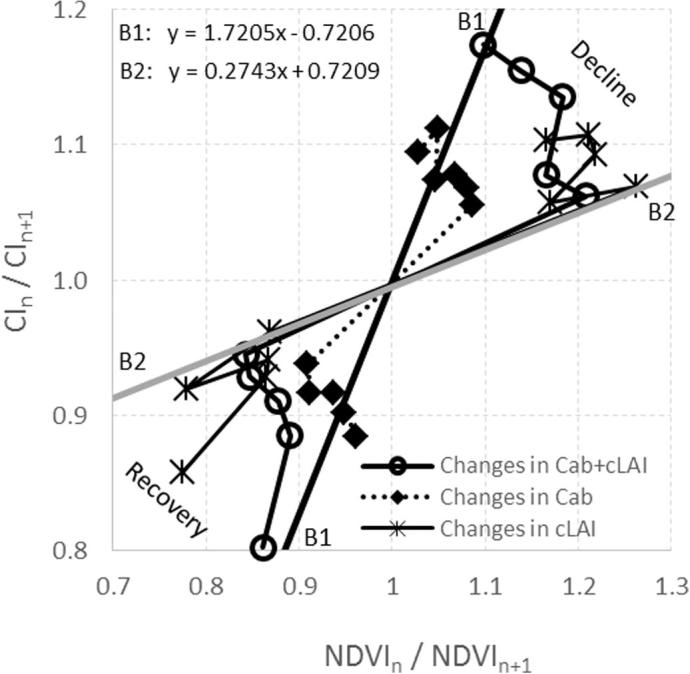
Trajectories generated in the subspace defined by the change rates of the CI vs. the NDVI for simulations of (i) a tree in which both C_ab_ and cLAI declined over time and later recovered (dots), (ii) a similar tree in which only C_ab_ changed (squares), and (iii) a similar tree in which only cLAI changed (stars). The arrows in the plot show the direction of the trajectory as trees undergo decline and then recover. The black (B1) and grey (B2) lines indicate the boundaries that establish the baselines for healthy and severely declining trees, respectively.

In fact, red-edge CI vs. NDVI trajectories corresponding to trees decreasing gradually in both C_ab_ and cLAI clearly moved away from those of healthy trees in a clockwise manner (Figs. 9d and 10). However, if both variables were decoupled and only one (chlorophyll or LAI) changed over time, the simulation results showed that chlorosis produced a clockwise change (Fig. 10a) while defoliation moved the trees away from the healthy ones along one of the boundaries (Fig. 10b). Furthermore, the temporal trajectories obtained for the CI vs. the NDVI accounting for changes into both decline and recovery (Fig. 11) showed different temporal trends between synthetic trees that underwent gradual changes in both C_ab_ and crown LAI and trees that underwent defoliation without pigment degradation and vice versa. The equations for the black (B1) and grey (B2) baselines indicated in Fig. 11 are the boundaries for healthy and severely declining trees, respectively.

**Fig. 12:**
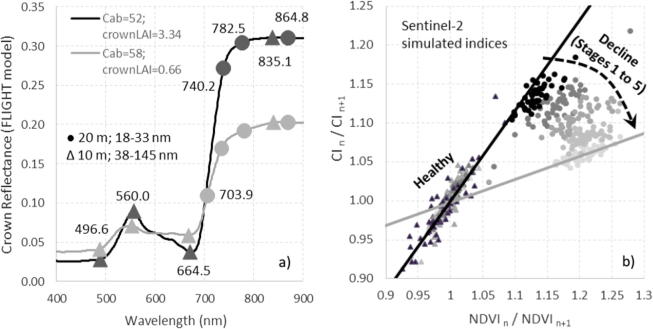
(a) Simulated crown reflectance for two trees, with the central wavelengths of the blue, green, red, NIR, and red-edge bands of the MultiSpectral Imager on Sentinel-2a; (b) Adaptation of the methodology proposed in this paper to Sentinel-2a data, showing change rates of the CI vs. the NDVI derived from model-generated spectra and using 665 nm and 835 nm bands for the NDVI, and 705 nm and 740 nm bands for the red-edge CI. On (b) triangles (dots) show the simulated healthy (decline) trees. Decline stages are shown in grey levels from black (low decline) to light grey (high decline).

Adapting the method to the spectral bands and bandwidths of the MultiSpectral Imager for calculating the NDVI and red-edge CI (Fig. 12a) from Sentinel-2a data enabled the definition of the healthy/decline baselines for this sensor based on the model simulation (Fig. 12b). Although the change of band set (hyperspectral vs. Sentinel-2a) affected the range of variation (compare Fig. 12b to Fig. 9d), the method is potentially applicable to Sentinel-2 data due to the sensitivity of the MSI red-edge bands to chlorophyll and LAI changes. The actual applicability of the proposed method will depend on the percentage of vegetation cover within the Sentinel-2 pixels (10 m for the NDVI bands; 20 m for the red-edge bands) and the degree of within-pixel homogeneity encountered in the forest decline process under study.

### 3.3 Application of the *temporal trend* modeling method to the hyperspectral imagery

**Fig. 13:**
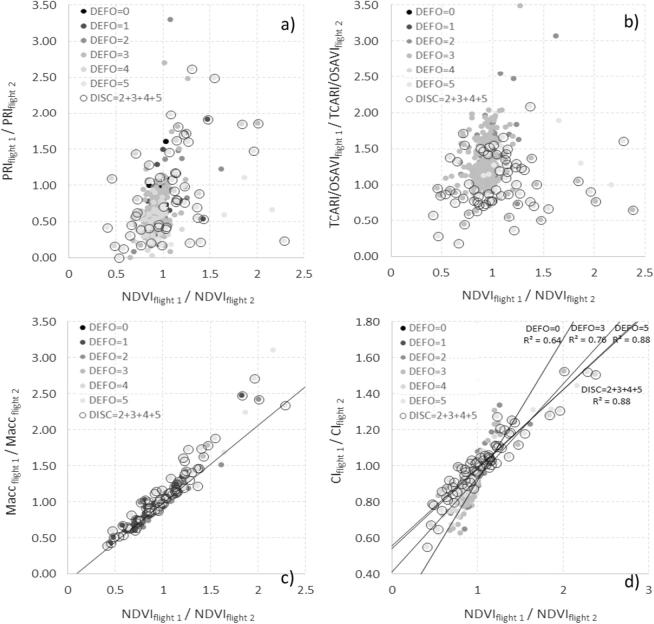
Application of the *temporal trend method* developed with synthetic data to real observations. Plots show for all trees assessed in the field for defoliation and discoloration the rate of change between two consecutive hyperspectral flights (acquired one year apart: January and December 2015), in terms of PRI (a), TCARI/OSAVI (b), Macc (c), and CI (d), vs. NDVI. The bottom right panel shows the regression lines fitted to the most and least healthy trees (DEFO = 0 and DEFO = 5, respectively), illustrating how the slope between the rates of change for the CI vs. the NDVI decreased as tree health declined. Level 0 corresponds to trees without any sign of declining health, while other scores reflect the percentage of the crown displaying poor health: level 1 = [1–25%], level 2 = [26–50%], level 3 = [51–75%], level 4 = [76–99%], level 5 = 100%.

Applying the proposed methodology to pair of images from consecutive hyperspectral fights (flights 1 and 2; winter flights one year apart) allowed us to evaluate the temporal change rate for each tree inspected in the field (Fig. 13) examining the index pairs NDVI vs. PRI (Fig. 13a), NDVI vs. TCARI/OSAVI (Fig. 13b), NDVI vs. Macc (Fig. 13c), and NDVI vs. CI (Fig. 13d).

**Fig. 14:**
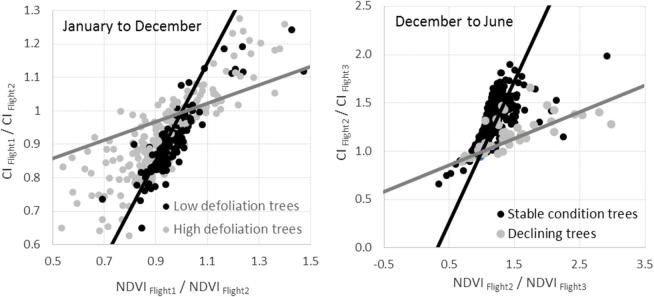
Left panel: CI change rates vs. NDVI change rates using data from hyperspectral Flight 1 (January 2014) and Flight 2 (December 2015) to distinguish between trees showing low and high levels of defoliation. Black and grey lines show the boundaries of the space occupied by the trees and set a baseline for healthy and declining trees, respectively, according to the model simulations (see Fig. 10). Right panel: CI change rates vs. NDVI change rates between hyperspectral Flight 2 (December 2015) and Flight 3 (June 2016) for trees identified in the field as being in stable condition between both dates and those whose health had declined. In the space defined by the two change rates, the former trees remained on the healthy baseline, established using Flight 1 and Flight 2 data, while the latter trees fell on the decline baseline.

The slope between the rates of change for CI vs. NDVI decreased as tree health declined (Fig. 13d). The other indices calculated from the hyperspectral imagery for the field-surveyed trees did not show consistent differences between trees in different health conditions. A more detailed view of the subspace created by the CI vs. NDVI change rate for the consecutive flights 1 and 2 (i.e. depicting changes from January to December) and flights 2 and 3 (changes from December to June) (Fig. 14) shows that healthy trees characterized by low defoliation fell within the same baseline (Fig. 14a), which is consistent with the modeling results (Fig. 9d); by contrast, trees undergoing some degree of decline fell on either side of the subspace, probably depending on whether their health was worsening or recovering.

To further validate these initial findings with field data, at the time of Flight 3 (June 2016) we revisited a subset of the trees inspected around Flight 2 (December 2015), recording whether they had maintained a stable condition or whether they had declined between the two dates. When these trees were positioned in the temporal change rate subspace (Fig. 14b) and were compared to the baselines established using the Flight 1 and Flight 2 data, stable trees tended to remain on the healthy baseline, while those undergoing decline from Flight 2 to Flight 3 aligned over the decline baseline, in agreement with the model-based predictions (Figs. 9d and 10a, b). The recovery trend observed in the theoretical model (Fig. 11) could not be validated with the dataset of this study since the area was heavily affected by a dominant decline, and in general there were no trees that followed a substantial recovery.

### 3.4 Application of the *temporal trend modeling method* to Sentinel-2a imagery

**Fig. 15:**
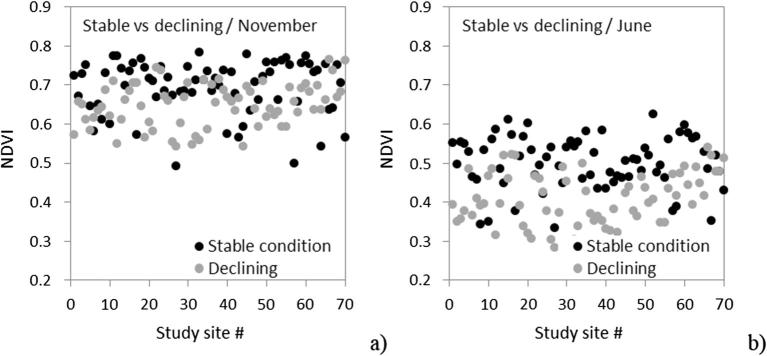
Sentinel-2a NDVI for pixels covering field-evaluated trees in winter (a) and summer (b) distinguishing healthy and declining trees, as determined by comparing crown status in June 2016 and November 2015. Sentinel-2a images were acquired on 29 November 2015 and 19 June 2016.

The very high resolution (40 cm) of the hyperspectral images enabled the identification of pure tree crowns and a direct comparison with the field assessments of decline. The application of the *temporal trend method* developed here to real Sentinel-2a images first required an assessment of the sensitivity of Sentinel-2 images, which have 10–20 m resolution, to the forest decline levels observed in the field for individual trees. We therefore identified each Sentinel-2 pixel covering a field-inspected tree and compared the NDVI to the CI (data not shown) for pixels covering healthy vs. declining trees in winter and summer (Fig. 15). The comparison revealed that Sentinel-2 images of NDVI captured the expected seasonal changes between winter (Fig. 15a) and summer (Fig. 15b) for pixels falling over trees under decline and stable health condition. Nevertheless, the seasonal and sun-angle effects observed between winter and summer were greater than the differences found between healthy and declining pixels in both seasons (Fig. 15).

**Fig. 16:**
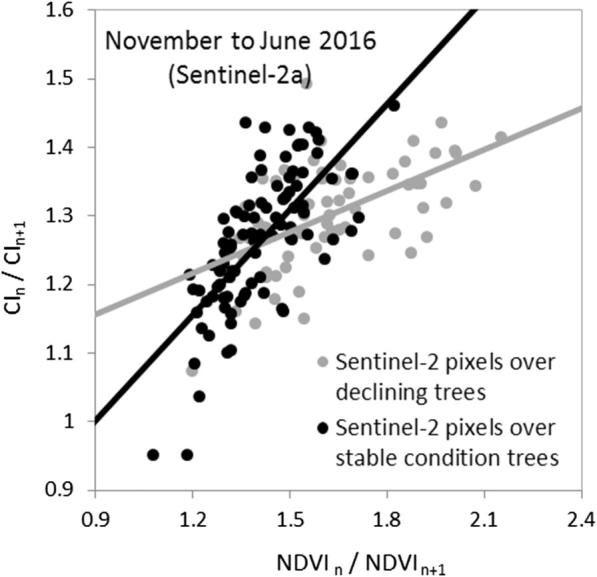
Temporal change rate based on November 2015 and June 2016 Sentinel-2a images for the NDVI structural index vs. the red-edge CI. Grey and black dots respectively represent pixels in the study area where trees in declining and healthy condition were identified during field campaigns.

We tested whether the methodology developed here and adapted to the Sentinel-2a band set (Fig. 12) made it possible to distinguish Sentinel-2 pixels with declining trees from those with only healthy trees despite the strong seasonal trends in the data (Fig. 15). The temporal rate of change of the Sentinel-2 NDVI vs. CI between both dates (December 2015 vs. June 2016) showed that the Sentinel-2 pixels with trees undergoing decline had a distinct trajectory from pixels with healthy trees (Fig. 16). The analytical method proposed here proved to be useful to detect ‘hotspots’ of stress or disturbance in coniferous forests using Sentinel-2 data. Nevertheless, issues regarding the spatial resolution and the effects of the background on the baselines derived to detect decline will require further study before this methodology can be operationally applied to Sentinel-2 data in other habitats or over very large areas. In particular, we show that the theoretically-derived baselines developed for tree-crown level resolution (Fig. 11) varied with respect to the ones shown for Sentinel-2a (Fig. 16) due to the background effects on the Sentinel-2 pixels. Therefore, specific baselines will need to be developed to properly account for the pixel aggregation by scene components in sparse canopies such as in the present study.

## 4 Conclusions

A radiative transfer modeling approach using PROSPECT and the 3D FLIGHT model demonstrated that the temporal rate of change of the NDVI structural index vs. the red-edge CI followed temporal trajectories associated with decline processes in pine trees. These models, used here to simulate a low-density coniferous forest undergoing decline through temporal changes of C_ab_ and crown LAI, showed that gradual decline processes affected the entire red-edge spectral region. Exploring different indices in the red edge, we found that the temporal change rate of the CI vs. the NDVI made it possible to distinguish trees in healthy conditions (simulated in synthetic spectra as random variation in C_ab_ and crown LAI) from those undergoing decline. Applying this *temporal trend method* to high-resolution hyperspectral images acquired for two years over a pine forest locally exhibiting chlorosis and defoliation processes demonstrated the following: pine trees under decline showed a different NDVI vs. CI temporal trajectory from that of trees in stable condition. In the temporal change rate relationship obtained for the NDVI vs. the CI, healthy trees concentrated along a single regression line, effectively establishing a baseline for healthy condition. By contrast, trees in very poor health concentrated on a different boundary, defined as the baseline for decline condition. Trees at progressive stages of crown decline – particularly defoliation – tended to gradually move from the healthy baseline to the decline baseline.

Trees that were assessed in the field as undergoing decline (i.e., observed to be in worse condition in June 2016 than in December 2015) aligned towards the decline baseline, in agreement with the modeling results. The application of this *temporal trend method* to real Sentinel-2a images coincident with the field observations showed that Sentinel-2 pixels falling over trees undergoing decline were also aligned towards the decline baseline. Our results obtained theoretically using radiative transfer models and confirmed empirically with high-resolution airborne hyperspectral and Sentinel-2 satellite images thus demonstrate the potential to use this *temporal trend method* based on the rate of change of the red edge to detect and monitor decline processes in trees and forests, even sparse ones.
